# The Molecular Characterization of Bovine Leukaemia Virus Isolates from Eastern Europe and Siberia and Its Impact on Phylogeny

**DOI:** 10.1371/journal.pone.0058705

**Published:** 2013-03-19

**Authors:** Marzena Rola-Łuszczak, Aneta Pluta, Monika Olech, Irina Donnik, Maxim Petropavlovskiy, Anton Gerilovych, Irina Vinogradova, Bhudipa Choudhury, Jacek Kuźmak

**Affiliations:** 1 Department of Biochemistry, National Veterinary Research Institute, Pulawy, Poland; 2 Urals State Scientific Research Institute of Veterinary Medicine, Ekaterinburg, Russia; 3 National Scientific Center 'Institute of Experimental and Clinical Veterinary Medicine, Kharkov, Ukraine; 4 All Russian Research Institute of Animal Breeding, Moscow region, Dubrovitsy, Russia; 5 Department of Virology, Animal Health and Veterinary Laboratories Agency (AHVLA), New Haw, Surrey, United Kingdom

## Abstract

Recent studies have shown that bovine leukemia virus (BLV) sequences can be classified into seven distinct genotypes based on full gp51 sequence. This classification was based on available sequence data that mainly represented the BLV population that is circulating in cattle from the US and South America. In order to aid with a global perspective inclusion of data from Eastern Europe is required. In this study we examined 44 BLV isolates from different geographical regions of Poland, Belarus, Ukraine, and Russia. Phylogenetic analysis based on a 444bp fragment of *env* gene revealed that most of isolates belonged to genotypes 4 and 7. Furthermore, we confirmed the existence of a new genotype, genotype 8, which was highly supported by phylogenetic computations. A significant number of amino acid substitutions were found in the sequences of the studied Eastern European isolates, of which 71% have not been described previously. The substitutions encompassed mainly the C-part of the CD4+ epitope, zinc binding peptide region, CD8+ T cell epitope, and overlapping linear epitope E. These observations highlight the use of sequence data to both elucidate phylogenetic relationships and the potential effect on serological detection of geographically diverse isolates.

## Introduction

Bovine leukaemia virus (BLV) belongs to the genus *Deltaretrovirus* of the *Retroviridae* family and it is etiologic agent for enzootic bovine leucosis (EBL) [1], [2]. In the course of infection the majority of BLV-infected cattle remain clinically healthy; however, about one-third of infected animals develop persistent lymphocytosis as a result of polyclonal proliferation of B lymphocytes, mainly CD5+ cells and 0.1 to 10% develop lymphoid tumors [3]. BLV serological surveys reveal that the infection is widely disseminated throughout the world with a high prevalence in North and South America, some Asiatic and Middle Eastern countries as well as Eastern Europe [4].

Like other complex retroviruses the BLV genome contains the *gag*, *pol* and *env* structural genes and the regulatory genes which includes the Tax, Rex, R3 and G4 [2], [5]. The *env* gene encodes the gp51 envelope and the gp30 transmembrane glycoproteins which are essential to confer viral infectivity and elicit the neutralising of the antibodies response [6].

Genetic variation of the *Deltaretrovirus* genus appears to be minimal as compared to that of human and animal lentiviruses [7], [8]. A full genome sequence comparison of three BLV isolates from Belgium, Japan and Australia revealed that they share 97% nucleotide homology [9]. An analysis focusing on genetic variations of gp51 sequence also demonstrated significant sequence conservation of BLV isolates from multiple geographical locations [10], [11]. These observations are in agreement with those previously made by Mamoun *et al* [12] and Beier *et al* [13] who demonstrated only small differences, mainly point mutations, within seven and twenty two geographically different strains respectively. These mutations conferred some differences in restriction enzyme sites allowing the classification of BLV isolates into three [14] and six [15] genotypes by RFLP analysis. It was also demonstrated that some genotypes can influence the leukomogenecity of a virus [16]. Fechner *et al* [14] reported the association between some BLV variants and the failure of antibody detection in infected cattle; however, Licursi *et al* [15] and Asfaw *et al* [17], did not observe a relationship between particular genotypes and the serological status of infected animals.

Characterization of the global BLV genetic diversity is an ongoing international research effort. In such studies, phylogenetic analysis were conducted showing that the *env*-derived sequences could be grouped into three [18] or four [11], [19] different genetic subgroups. A study by Rodriguez *et al* [20] which integrated the available full gp51 sequences of BLV from different geographic origins, clearly showed that BLV sequences could be classified into seven distinct genotypes. However, as this classification was based on available sequence data, whilst it included sequences from Asia, Europe and Australia, it mainly represented the BLV circulating in cattle from the Americas.

There is a lack of comprehensive studies focusing on genetic characterization and classification of BLV isolates present in Eastern Europe and Russia. In this paper we attempt to address this by discussing the characterization and phylogenetic analysis of *env* gene sequences from 44 BLV isolates from different geographical regions of Poland, Belarus, Ukraine and Russia.

## Materials and Methods

### Samples origin

Blood samples were collected from 44 cattle, naturally infected with BLV and serologically positive, as was diagnosed by ELISA (Institut Pourquier blocking ELISA, Synbiotic Monoblocking ELISA) or AGID (agar gel immunodiffusion) tests. These animals came from 14 herds, located in 13 geographically distinct regions within four countries: Poland, Ukraine, Belarus and Russia. The distribution and location of these regions is shown in Table S1 and in the herd map -Figure S1. Blood samples from Russia and Ukraine were originally collected by collaborating laboratories from these countries and then sent to the National Veterinary Research Institute in Pulawy in the form of dry pellets of peripheral blood leukocytes (PBL) or as lyophilisated DNA samples. Blood samples from Poland were selected by national reference laboratory during EBL monitoring programme. Two archived samples from BLV positive cows were received from national reference laboratory in Minsk, Belarus.

### DNA isolation

PBLs were isolated by centrifugation at 1500 g for 25 min and erythrocytes were haemolysed by osmotic shock with H_2_O and 4.5% NaCl. After two washes in PBS, the supernatant was discarded and the cell pellet (5×10^6^ cells) was used for extraction of genomic DNA with the DNeasy Tissue Kit (Qiagen) following manufacturer’s recommendations. DNA concentration was calculated (Nanophotometer) and the samples were stored at – 20°C until PCR amplification.

### PCR amplification of 444bp env gene fragment

The primers for nested PCR amplification were described previously by Beier *et al* [13] their sequence is as follows: *env* 5032 (5′-TCTGTGCCAAGTCTCCCAGATA-3′); *env* 5608 (5′-AACAACAACCTCTGGGAAGGGT-3′) and e*nv* 5099 (5′-CCCACAAGGGCG GCGCCGGTTT-3′), *env* 5521 (5′-GCGAGGCCGGGTCCAGAGCTGG-3′). Amplification was performed with 500 ng of genomic DNA using Thermal Cycler (Biometra) with the following cycling conditions: 2 min at 94°C, 30 s at 95°C, 30 s at 62°C (external primers) or 30 s at 70°C (internal primers), 1 min at 72°C; after the last (40^th^) cycle, the samples were incubated at 72°C for 4 min. Each 50 μl reaction contained 5 µl 10x buffer, 1 µl of 10 mM dNTPs, 1.5 µl of 10 mM of each primer and 1 µl of DyNAzymeTMII DNA Polymerase (Finnzymes). After amplification, PCR products from each sample were separated and analysed by electrophoresis on 1.5% agarose gel containing ethidium bromide (1 µg/ml) in 1x TAE buffer.

### DNA sequencing and analysis

PCR products were purified using the NucleoSpin Extract II kit (Marcherey-Nagel GmbH & Co). The purified amplicons were sequenced on 3730xl DNA Analyzer (Applied Biosystems) using Big Dye Terminator v3.1 Cycle Sequencing Kit. Sequence data were analysed using the BioEdit sequence alignment editor and subsequently were aligned using the Geneious Alignment module within Geneious Pro 5.3 Software (Biomatters Ltd) [21]. The resultant 44 sequences (400 bp size after subtracting the length of the primers sequence) were submitted to GenBank and assigned accession numbers as documented in Table S1. In addition to the 44 BLV sequences generated in this study, further publicly available sequences representing the eight known genotypes were included for analysis (Table S1). Phylogenetic analysis was performed using the tree-builder tool of the Geneious software. Phylogenetic trees were constructed using Mr Bayes method with GTR substitution model and the neighbour-joining (NJ) method [22] with Tamura-Nei model of nucleotide substitution [23] with the robustness of the clusters assessed by bootstrapping 1000 replicates. Mean nucleotide distances within (intra-genotype) and among (inter-genotype) BLV genotypes were estimated by adopting the Tamura Nei model in MEGA 4 [24].

## Results

### Phylogenetic analysis of BLV isolates

**Figure 1:**
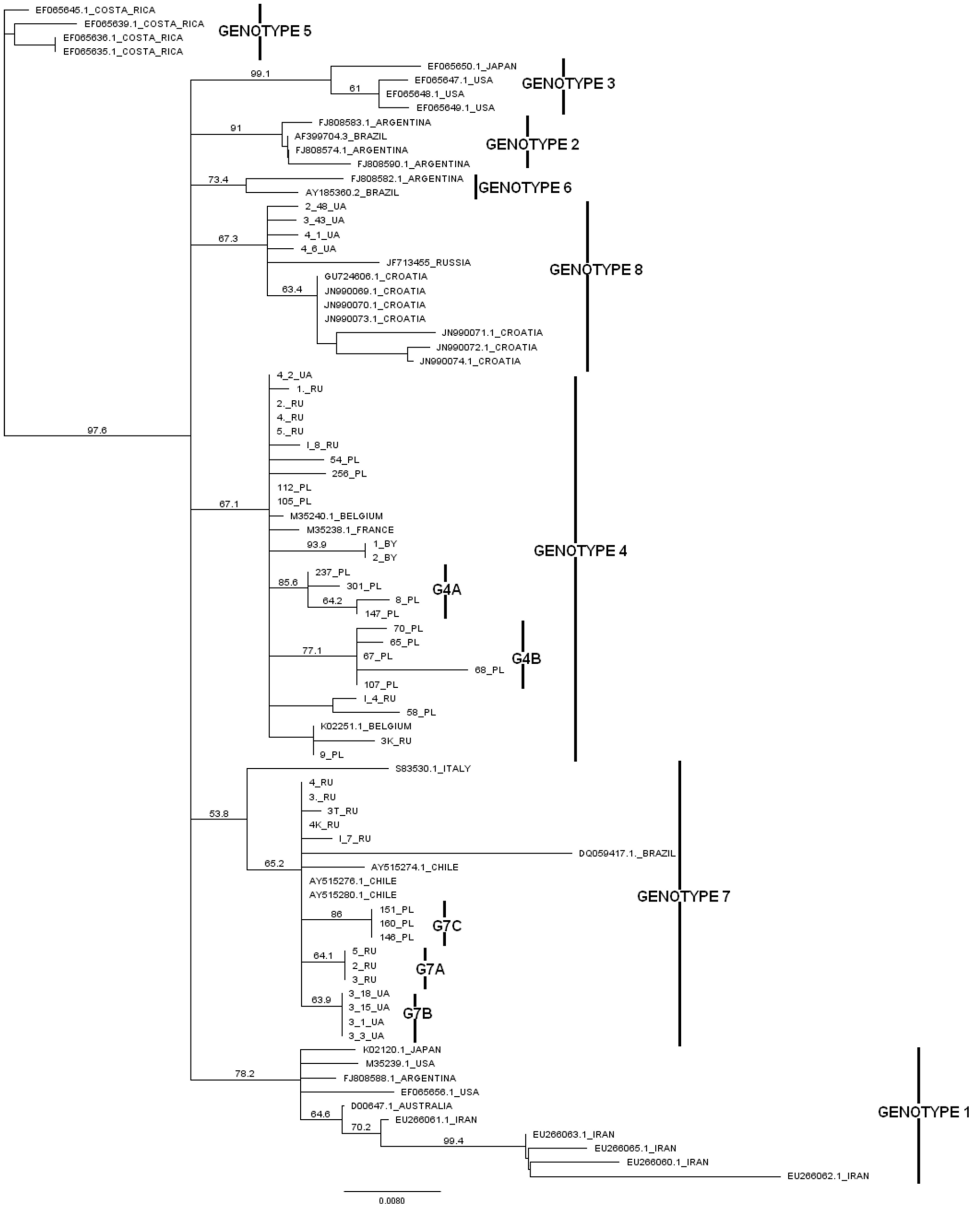
Neighbour-joining phylogenetic tree based on 400 bp of *env* gene sequences of BLV isolates. Euroasian isolates are shown by name. Remaining strains in the tree are shown by accession number and country of origin. Numbers at the branches show bootstrap support. Genotypes are indicated by vertical line.

**Figure 2:**
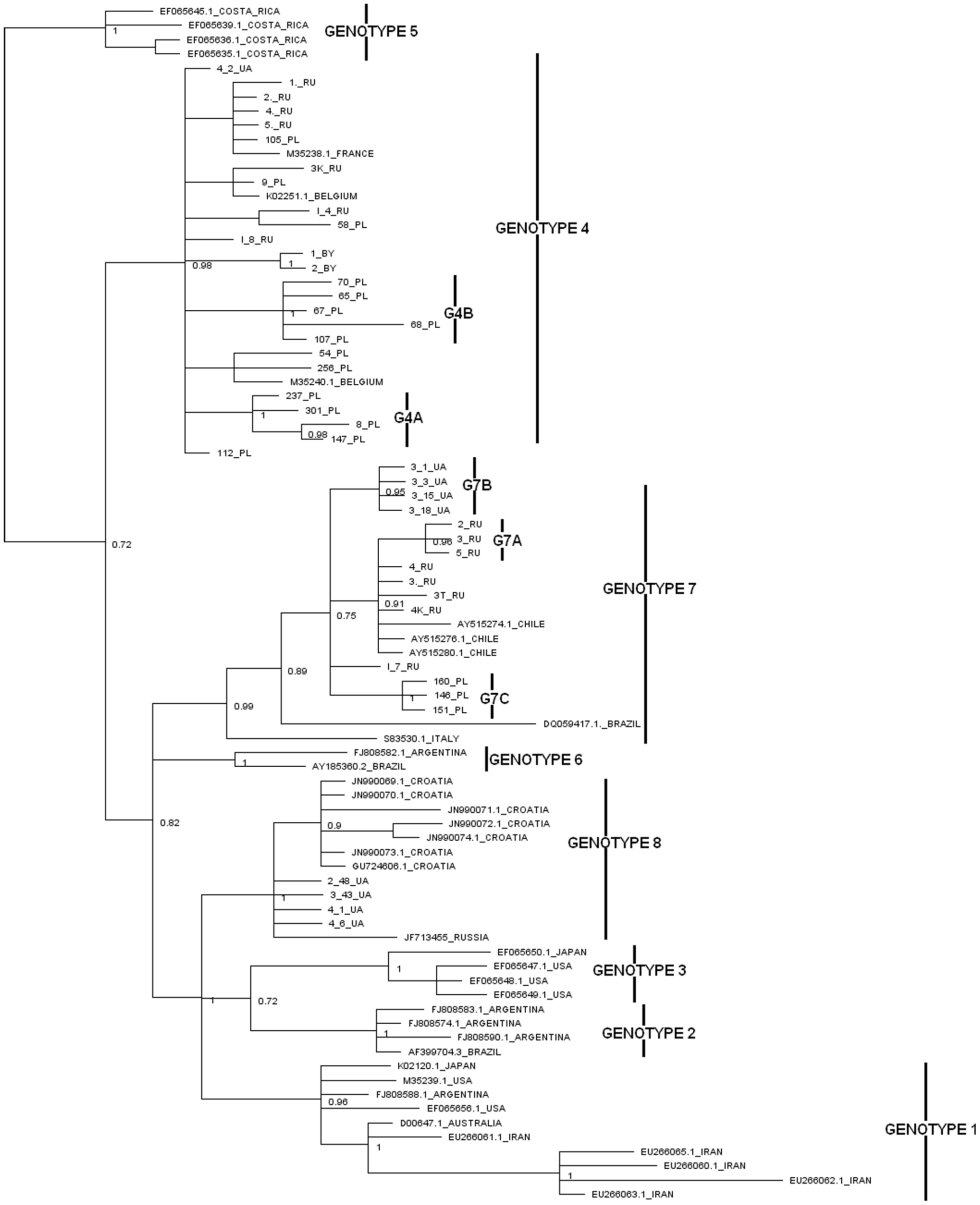
Bayesian phylogenetic tree based on 400 bp of *env* gene sequences of BLV isolates. Euroasian isolates are shown by name. Remaining strains in the tree are shown by accession number and country of origin. Numbers on nodes indicate posterior probabilities. Genotypes are indicated by vertical line.

All 44 DNA samples which comprised of 15 Russian, 18 Polish, 9 Ukrainian and 2 Belarusian cattle were subjected to nested PCR amplification and a 444 bp fragment of *env* gene was successfully amplified and sequenced from all samples. In order to analyse the degree of genetic variability of these isolates respective sequences were aligned with 38 other sequences from GenBank representing the eight described genotypes. We aimed to include as many geographically diverse strains as possible however we only included sequences which were at least 400bp in length to match data generated in our laboratory. These comprised BLV isolates from USA, Argentina, Brazil, Chile, Costa Rica, Croatia, Iran, Australia, Japan and Western Europe, which were recently classified by Rodriguez *et al* , Moratorio *et al* and Balic *et al* [20], [25], [26]. Additionally, we used two sequences (GU724606.1, JF713455.1) from Croatia and Russia respectively, which have not yet been investigated by published phylogenetic analysis. Using neighbour-joining (NJ) and Bayesian Markov chain Monte Carlo methods, based on nucleotide sequence alignment of 400 bp length part of the *env* gene, phylogenetic trees were constructed as shown in Figures 1 and 2 respectively. When NJ phylogeny was performed 40 out of 44 analysed sequences clustered with either genotype 4 (G4) or 7 (G7) sequences, with 67.1% and 53.8% bootstrap proportions support respectively. Four sequences, representing Ukrainian cattle (3_43_UA, 4_1_UA, 4_6_UA, 2_48_UA) as well as sequences JF713455.1 and GU724606.1 clustered within the recently identified - new BLV genotype with 67.3% bootstrap value.

To validate the data of NJ analysis the Bayesian method was applied. As shown in Figure 2, a set of 40 sequences were distributed within G4 and G7, which was supported by the high posterior probabilities of 0.98 and 0.99 , respectively. Four remaining strains from Ukraine, one from Croatia (GU724606.1) and one from Russia (JF713455.1) could again not be assigned to any of already known seven genotypes and clustered with isolates described by Balic *et al* [26]. As shown in Figures 1 and 2 there is significant support for this new genetic group with values 1 and 67,3% of Bayesian and NJ analysis, respectively.

Since 40 out of 44 isolates were identified within G4 and G7, their distribution to country origin and herd localisation was analysed. G7 included 15 out of 40 analysed sequences of which 8, 4 and 3 sequences were represented by Russian, Ukrainian and Polish isolates respectively. These isolates were segregated into five herds from Russia, one herd from Ukraine and one herd from Poland. Furthermore, phylogenetic analysis clearly showed the existence of three separated subgroups within G7, whether the tree topologies were derived from NJ or Bayesian methods. Since their existence was supported by a significant branch support (posterior probabilities from 0.95 to 1.00 and bootstrap values from 63.9% to 86%) we propose to distinguish three subgroups within the seventh genotype labelled G7A, G7B and G7C on Figures 1 and 2. Subgroup G7A was comprised exclusively of Russian isolates, while subgroups G7B and G7C included isolates from Ukraine and Poland, respectively. These subgroups had the significant branch support reflecting variability within genotype G7, with a maximum divergence of 3.1% of nucleotide sequence. Furthermore, the existence of distinct subgroups within G7 fully corresponded to the geographical origin of the BLV isolates, since each particular subgroup contained isolates from one country only. However, the rest of the five isolates from Russia are localised close to those from Chile; therefore it is not known whether including further isolates from these countries would alter this finding.

G4 included 25 out of 40 analysed sequences, with 7, 1, 15 and 2 sequences representing Russian, Ukrainian, Polish and Belarusian isolates respectively. These isolates came from three herds from Russia, one from Ukraine, six from Poland and one from Belarus. Sequence analysis revealed that the sequence divergence within G4 was similar (2.8%) as to that noted for G7. In the G4 subgroup distribution was also visible, particularly among two subgroups labeled as G4A and G4B in figures 1 and 2, representing exclusively the BLV isolates of Polish origin. It was supported with a high (1.0) value of posterior probabilities and bootstrap value of 85.6% (G4A) and 77.1% (G4B). The same high posterior probabilities were noted for subgroup consisting of two isolates from Belarus; however, it was rather difficult to distinguish a separate subgroup based on the limited number of isolates.

As indicated above, both NJ and Bayesian trees confirmed the existence of a new genotype: G8. To confirm that G8 clearly creates a distinct cluster, the mean intra- and inter-genotype genetic distances were calculated. As shown in Table 1 the mean distances between isolates (intra-genotype) belonging to G8 were not significantly different from those observed within G1–G7, while the mean nucleotide distances (inter-genotype) between G8 and remaining genotypes were comparable to each other and furthermore one order of magnitude higher than the mean intra-genotype distances. Thus, we additionally proved that a new genotype 8 creates a distinct cluster within BLV phylogeny. In our study, G8 included four isolates from cattle from three geographically distinct regions of Ukraine and isolates from Croatia (GU724606.1) and Russia (JF713455.1). The pair-wise genetic distances analysis within this genotype ranged between 0.0 – 2.3% and so it was relatively lower than those observed for G4 and G7. Surprisingly, this low level of genetic diversity did not reflect the broad geographical origin of these isolates, perhaps indicating the same origin of infection.

**Table 1:** Mean nucleotide distances in 400 bp fragment of envelope gene within (intra-genotype) and among (inter-genotype) BLV genotypes.

	G1	G2	G3	G4	G5	G6	G7	G8
G1	0.021 ± 0.004 *	–	–	–	–	–	–	–
G2	0.037 ± 0.008	0.004 ± 0.002 *	–	–	–	–	–	–
G3	0.041 ± 0.008	0.028 ± 0.008	0.008 ± 0.003 *	–	–	–	–	–
G4	0.039 ± 0.008	0.025 ± 0.007	0.035 ± 0.008	0.011 ± 0.002 *	–	–	–	–
G5	0.044 ± 0.009	0.036 ± 0.009	0.043 ± 0.010	0.033 ± 0.008	0.005 ± 0.003 *	–	–	–
G6	0.035 ± 0.008	0.026 ± 0.007	0.034 ± 0.008	0.025 ± 0.006	0.037 ± 0.009	0.013 ± 0.005 *	–	–
G7	0.040 ± 0.008	0.027 ± 0.008	0.035 ± 0.008	0.029 ± 0.007	0.035 ± 0.008	0.028 ± 0.007	0.010 ± 0.002 *	–
G8	0.033 ± 0.007	0.025 ± 0.007	0.028 ± 0.007	0.027 ± 0.007	0.037 ± 0.009	0.025 ± 0.007	0.028 ± 0.007	0.008 ± 0.002 *

* mean intra-genotype nucleotide distances

### Amino acid sequences analysis

**Figure 3:**
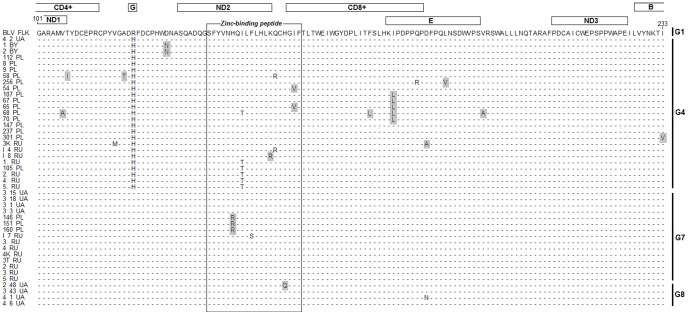
Alignment of the partial amino acid *env* sequences from 44 BLV isolates. All newly found substitutions are marked with light grey fields. The labeled rectangles in the upper part of figure refer to the coding sequences of antigenic determinants and the black frame on alignment refer to the Zinc-binding peptide. The localization of 4, 7 and 8 BLV genotypes is represented by the black bars in the right part of figure. Dots indicate identity with BLV_FLK (M35242.1), which was used as reference in this work.

We analysed whether nucleotide mutations affected amino acid composition.. Amino acid (aa) sequences of the 44 BLV isolates were aligned to aa sequence of BLV-FLK (M35242.1). Figure 3 represents the distribution of amino acid changes within middle portion of gp51 glycoprotein, encompassing amino acids at position 101 to 233. We noted 21 different amino acids substitutions. Furthermore, although a variety of single amino acid substitutions were evident over the full length of the analysed part of gp51 some amino acids changes (R121H, H142R, I144T, I176L) were observed in multiple samples. These substitutions encompassed mainly the C-part of CD4+ epitope, zinc binding peptide region, CD8+ T cell epitope and overlapping linear epitope E. Another feature evident from this alignment is that the highest numbers of amino acid substitutions were observed in isolates belonging to G4.

## Discussion

According to previously published phylogenetic data, BLV isolates collected in different geographical locations showed a correlation between genotype grouping and their geographical origin [20]. The main branch of G1 is represented by isolates from US, Japan and South American countries with a separate cluster containing Australian and Iranian BLV strains. G3 has isolates from US and Japan. Uniquely, G2 and G5 were exclusively composed from Argentinean and Costa Rican isolates. The most common genotype in Europe appears to be G4, although as whole these are clustered a few representatives from Argentina. G6 includes sequences from Argentina and Brazil [20]. Until recently, the Italian strain (GenBank accession: S83530) was the only member of genotype G7 until Chilean strains were also assigned there [25]

The phylogenetic analysis presented in this paper concurs with previously established groups as well as discovering unknown affiliations between newly described sequences. Namely, all 44 BLV sequences of Eastern European and Russian origin, including isolates from Poland, Russia, Ukraine and Belarus were assigned to three genotypes: G4, G7 and a new genotype, G8. We found that most of these isolates (25/44) clustered within G4. BLV strains were distributed rather randomly, although certain clustering could be observed with reference to Polish sequences (G4A and G4B in Figure 1 and Figure 2). All of these isolates originated from distant regions of the four countries which were located at long distances away from each other, around four thousand kilometers. Furthermore, all of them were clustered together with isolates that originally were identified in France and Belgium. Since a virus’s introduction to a herd via the import of infected cattle is considered as a main route of transmission of BLV infection [11], [27] we propose that localisation of BLV strains in genotype G4 covering such a large geographic area can be explained by extensive cattle trading. During World War II approximately 70% of the cattle population in Poland was destroyed and within the framework of reparations and during the post-World War II development, cattle were imported from western European countries, including amongst others Germany, Netherlands and Denmark. Similarly in the early 1960s, import of live cattle from Denmark was reported in the former Soviet Union. It seems plausible that the post-war expansion of the former Soviet Union might have created an environment that allowed the broad transmission of the BLV. Indeed, extensive livestock trade took place between countries belonging to CMEA (Council for Mutual Economic Assistance) in the 1970s and 1980s. Therefore, the topology of the G4 genotype could be explained by animal trade.

Fifteen BLV isolates were classified in G7, and for the first time we showed the extended topology of this genotype. G7 contains isolates from Italy and Brazil located on separate branches and Chilean isolates closely related to isolates from Eastern Europe and Siberia. In contrary to the topology of G4, where the BLV strains were distributed rather randomly, the distribution patterns within G7 showed the existence of three well defined subgroups. Each of these subgroups exclusively contained isolates from Russia (G7A), Ukraine (G7B) and Poland (G7C). Identification of the origin of these isolates showed that they came from one particular region of each of these countries; therefore, it is not surprise that they clustered according to the place of origin. The presence of such region or herd tailored geno-grouping of BLV isolates can occur as a results of transmission of a fraction of the virus population. This could reinforce the concept of a role for geographical isolation in BLV diversification, possible associated to genetic drift. Effects of genetic drift probably caused virus assimilation within their populations, as in the case of the virus populations G7A, G7B, G7C. Over time these small populations became homogeneous. An example of this may be the presence of a single amino acid mutation H142R, which is unique and specific for G7C subgroup. It may be the case that in such a small population a mutation such H142R rapidly became perpetuated as a permanent mutation.

Our study also confirmed the existence of the new genotype: G8. This finding was supported not only by NJ and Bayesian analysis but also by intra- and inter-genotype genetic distance analysis. The existence of this new genotype was noticed previously by Matsumura *et al* [28], however, this data could not be validated since the classification of isolates was conducted by the UPGMA method. It has been shown that UPGMA is extremely sensitive to unequal rate in different lineages and also can not detect zero length branches therefore this method is not considered as suitable for phylogenetic analysis as other methods [29], [30]. G8 comprised of four isolates from Ukraine, one isolate from Russia and seven from Croatia representing different geographical locations: whilst most of the other Ukrainian isolates were dispersed within G4 and G7 the four isolates that clustered within G8 were closely related to each other as were the seven Croatian which created completely separate node. These cluster again emphasized correlation between grouping and geographical origin of BLV isolates.

In this study we also aligned deduced amino acid sequence of 44 isolates to that of gp51 protein of BLV FLK. Many amino acid changes were present in sequences of almost all the studied Eastern European isolates and they were confined to four regions: C-part of CD4+ epitope, zinc binding peptide region, CD8+ T cell epitope and also overlapping epitope E. It was interesting that more than half (71%) of all amino acid substitutions reported in this study have not been described previously [20], [25], [28]. The biological significance of these changes is unknown, although the histidine replacement (H) by arginine (R) at position 142 could be speculated. In some BLV isolates this histidine was replaced by tyrosine (Y) or leucine (L) [20] and it was shown that this histidine is one of the three histidine residues which are present in the zinc-binding region, which is an essential component of zinc-binding proteins together with cysteine. Taking into account that the region of SU localised between residues 137–156 affects fusion and infectivity of BLV *in vivo* [31] this mutation may be crucial for infectivity. This prediction could be supported by work conducted by Zavorotinskaya *et al* [32] who found that the aromatic side chain of histidine plays a critical role in Env-mediated fusion of ecotropic murine leukaemia virus (MLV).

We also noted some amino acid substitutions described in previous studies. The substitution of threonine (T) in place of isoleucine (I) at position 144 was observed in six isolates, which clustered G4. It has been shown that this change was present in the variant of BLV – LB59 (M35238), found in naturally infected cattle and it was assumed that this mutation together with substitutions of serine to phenylalanine at position 56 can influence epitope-H specific antibody recognition [33]. Furthermore, the substitution phenylalanine to serine at position 146, previously noted by Moratorio *et al* [25], was found in one isolate and according to predicted tertiary structure of gp51 this substitution is exposed on the surface of the second neutralizing epitope, thereby potentially diminishing immunoreactivity of this epitope.

Studying the genetic diversity of viruses could help to gain the correlation between variation in genotype and disease progression, differences in infectivity or potential effect of viral variability on diagnostic assays. We could speculate some differences in infectivity between described BLV strains based on observed mutations as was previously hypothetized by other researches [34], [35]. However these can be only predictions due to the lack of data from testing the infulence of these mutations on the viral infectivity *in vitro* and *in vivo*. We also noted some amino acids modifications in the sequence of CD8+/epitope E region but they did not seem to influence of detection capability of serological diagnostic methods because all our samples were strongly positive both by ELISA/AGID and PCR assays. However observed nucleotide mutations on analysed 400 bp of *env* gene undoubtedly have to be considered during designing primers or probes for BLV amplifications and BLV vaccine design.

In summary, our study based on an analysis of 44 isolates showed that BLV strains infecting cattle from a large geographical area including Poland, Belarus, Russia, and Ukraine were characterized by a high degree of genetic variability; however, they can be classified within three genotypes. This is contrary to the topology of BLV isolates from the South American region, which are clustered with seven different BLV genotypes [25]. In order to gain a better understanding of BLV global genetic diversity, it is essential to include as many geographically diverse isolates as possible. We addressed this issue by the analysis of Eastern European BLV sequences and making these data public so they can be included by others in their analysis. We expect further clarity to sub-clades and perhaps even the discovery of more novel genotypes as further isolates are characterized. This highlights one of the limiting factors in the study of BLV: essentially the lack of sequence data which as can be seen here is not only important for elucidating phylogenetic relationships but also in highlighting potential effects on serological detection.

## Supporting Information

Figure S1 **Geographical distribution of 14 herds sampled in the study.**
(TIF)

Table S1 Identity and origin of the sequences analysed in the study.
(PDF)
