# Supplementary material for: The Molecular Characterization of Bovine Leukaemia Virus Isolates from Eastern Europe and Siberia and Its Impact on Phylogeny
Source: PLoS One. 2013 Mar 19;8(3):e58705. doi: 10.1371/journal.pone.0058705 (PMC3602460; doi:10.1371/journal.pone.0058705)
Supplement: Table S1 — Identity and origin of the sequences analysed in the study. (PDF) [file pone.0058705.s002.pdf]

Table S1. Identity and origin of the sequences analyzed in the study

Ordinal no	GenBank accession no	Geographic origin	Genotype*	Identity code & source
1	HM563768	Kirovohradska Oblast, Ukraine	8	Sample 2_48_UA, this work
2	HM563761	Kharkivska Oblast, Ukraine	7	Sample 3_1_UA, this work
3	HM563760	Kharkivska Oblast, Ukraine	7	Sample 3_3_UA, this work
4	HM563758	Kharkivska Oblast, Ukraine	7	Sample 3_15_UA, this work
5	HM563759	Kharkivska Oblast, Ukraine	7	Sample 3_18_UA, this work
6	HM563767	Kharkivska Oblast, Ukraine	8	Sample 3_43_UA, this work
7	HM563766	Rivnenska Oblast, Ukraine	8	Sample 4_1_UA, this work
8	HM563781	Rivnenska Oblast, Ukraine	4	Sample 4_2_UA, this work
9	HM563764	Rivnenska Oblast, Ukraine	8	Sample 4_6_UA, this work
10	HM563750	Sverdlovsk Oblast, Russia	7	Sample 2_RU, this work
11	HM563748	Sverdlovsk Oblast, Russia	7	Sample 3_RU, this work
12	HM563752	Sverdlovsk Oblast, Russia	7	Sample 4_RU, this work
13	HM563749	Sverdlovsk Oblast, Russia	7	Sample 5_RU, this work
14	HM563773	Krasnodar Territory, Russia	4	Sample 1.RU, this work
15	HM563772	Krasnodar Territory, Russia	4	Sample 2.RU, this work
16	HM563751	Krasnodar Territory, Russia	7	Sample 3.RU, this work
17	HM563770	Krasnodar Territory, Russia	4	Sample 4.RU, this work
18	HM563771	Krasnodar Territory, Russia	4	Sample 5.RU, this work
19	HM563756	Tyumen area, Russia	7	Sample 3T_RU, this work
20	HM563782	Kurgan area, Russia	4	Sample 3K_RU, this work
21	HM563753	Kurgan area, Russia	7	Sample 4K_RU, this work
22	HQ902260	Moscow area, Russia	4	Sample I_4_RU, this work
23	HQ902261	Moscow area, Russia	7	Sample I_7_RU, this work
24	HQ902262	Moscow area, Russia	4	Sample I_8_RU, this work
25	HQ902258	Belarus	4	Sample 1_BY, this work
26	HQ902259	Belarus	4	Sample 2_BY, this work
27	EU262579.1	Greater Poland, Poland	4	Sample 58_PL, this work
28	HM563778	Greater Poland, Poland	4	Sample 70_PL, this work
29	EU262578.1	Greater Poland, Poland	4	Sample 54_PL, this work
30	HM563776	Greater Poland, Poland	4	Sample 65_PL, this work
31	HM563775	Greater Poland, Poland	4	Sample 67_PL, this work
32	HM563774	Greater Poland, Poland	4	Sample 68_PL, this work
33	EU262584.1	Opole, Poland	4	Sample 256_PL, this work
34	EU262583.1	Opole, Poland	4	Sample 237_PL, this work
35	EU262575.1	Opole, Poland	4	Sample 301_PL, this work
36	EU262581.1	Pomeranian, Poland, 1st herd	4	Sample 112_PL, this work
37	EU262580.1	Pomeranian, Poland, 1st herd	4	Sample 105_PL, this work
38	HM563755	Pomeranian, Poland, 1st herd	4	Sample 107_PL, this work
39	EU262577.1	Pomeranian, Poland, 2nd herd	4	Sample 9_PL, this work
40	EU262576.1	Pomeranian, Poland, 2nd herd	4	Sample 8_PL, this work
41	HM563763	West Pomeranian, Poland	7	Sample 160_PL, this work
42	EU262555.1	West Pomeranian, Poland	7	Sample 151_PL, this work
43	EU262554.1	West Pomeranian, Poland	7	Sample 146_PL, this work
44	EU262582.1	West Pomeranian, Poland	4	Sample 147_PL, this work
45	JF713455	Russia	8	Shaeva et al. (2011) ‡
46	GU724606.1	Croatia	8	Lojkic (2010) ‡
47	EF065645.1	Costa Rica	5	[1]
48	EF065639.1	Costa Rica	5	[1]
49	EF065636.1	Costa Rica	5	[1]
50	EF065635.1	Costa Rica	5	[1]
51	FJ808582.1	Argentina	6	[2]
52	AY185360.2	Brazil	6	Camargos et al. (2004) ‡
53	EF065650.1	Japan	3	[1]
54	EF065647.1	USA	3	[1]
55	EF065648.1	USA	3	[1]
56	EF065649.1	USA	3	[1]
57	FJ808583.1	Argentina	2	[2]

58	FJ808574.1	Argentina	2	[2]
59	FJ808590.1	Argentina	2	[2]
60	AF399704.3	Brazil	2	Camargos et al. (2004) ‡
61	K02120.1	Japan	1	[3]
62	M35239.1	USA	1	[4]
63	D00647.1	Australia	1	[5]
64	FJ808588.1	Argentina	1	[2]
65	EF065656.1	USA	1	[1]
66	EU266060.1	Iran	1	[6]
67	EU266061.1	Iran	1	[6]
68	EU266062.1	Iran	1	[6]
69	EU266063.1	Iran	1	[6]
70	EU266065.1	Iran	1	[6]
71	S83530.1	Italy	7	[7]
72	M35240.1	Belgium	4	[4]
73	K02251.1	Belgium	4	[8]
74	M35238	France	4	[4]
75	DQ059417	Brazil	7	[9]†
76	AY515274	Chile	7	[10]
77	AY515276	Chile	7	[10]
78	AY515280	Chile	7	[10]
79	JN990069	Croatia	8	[11]
80	JN990070	Croatia	8	[11]
81	JN990071	Croatia	8	[11]
82	JN990072	Croatia	8	[11]
83	JN990073	Croatia	8	[11]
84	JN990074	Croatia	8	[11]

* Ordinal no. 1-44 genotypes identified in this work through phylogenetic analysis; ordinal no. 45- 74 genotypes identified by Rodriguez *et al* [2].
‡ Camargos *et al* 2004; Shaeva *et al* 2011 and Lojkic 2010, unpublished, direct submission to GenBank.
†Ikuno *et al* 2005, unpublished, presented and classified by Moratorio *et al* 2010 [9].

Appendix References

1. Zhao X, Buehring GC (2007) Natural genetic variations in bovine leukemia virus envelope gene: possible effects of selection and escape. Virology 366: 150–165.
2. Rodriguez SM, Golemba MD, Campos RH, Trono K, Jones LR (2009) Bovine leukemia virus can be classified into seven genotypes: evidence for the existence of two novel clades. J Gen Virol 90: 2788–2797.
3. Sagata N, Yasunaga T, Tsuzuku-Kawamura J, Ohishi K, Ogawa Y, et al. (1985) Complete nucleotide sequence of the genome of bovine leukemia virus: its evolutionary relationship to other retroviruses. Proc Natl Acad Sci U S A 82: 677–681.
4. Mamoun RZ, Morisson M, Rebeyrotte N, Busetta B, Couez D, et al. (1990) Sequence variability of bovine leukemia virus env gene and its relevance to the structure and antigenicity of the glycoproteins. J Virol 64: 4180–4188.
5. Coulston J, Naif H, Brandon R, Kumar S, Khan S, et al. (1990) Molecular cloning and sequencing of an Australian isolate of proviral bovine leukaemia virus DNA: comparison with other isolates. J Gen Virol 71: 1737–1746.
6. Hemmatzadeh F (2007) Sequencing and phylogenetic analysis of gp51 gene of bovine leukaemia virus in Iranian isolates. Vet Res Commun 31: 783-789.
7. Molteni E, Agresti A, Meneveri R, Marozzi A, Malcovati M, et al. (1996) Molecular characterization of a variant of proviral bovine leukaemia virus (BLV). Zentralbl Veterinarmed B 43: 201–211.
8. Rice NR, Stephens RM, Couez D, Deschamps J, Kettmann R, et al. (1984) The nucleotide sequence of the env gene and post-env region of bovine leukemia virus. Virology 138: 82–93.

9. Moratorio G, Obal G, Dubra A, Correa A, Bianchi S, et al. (2010) Phylogenetic analysis of bovine leukemia viruses isolated in South America reveals diversification in seven distinct genotypes. Arch Virol 155: 481-489.
10. Felmer R, Munoz G, Zuniga J, Recabal M (2005) Molecular analysis of a 444 bp fragment of the bovine leukaemia virus gp51 *env* gene reveals a high frequency of non-silent point mutations and suggests the presence of two subgroups of BLV in Chile. Vet Microbiol 108: 39–47.
11. Balic D, Lojkic I., Periskic M, Bedekovic T, Jungic A, et al. (2012) Identification of a new genotype of bovine leukemia virus . Arch Virol 157: 1281-1290.
